# Plant biologists FRET over stress

**DOI:** 10.7554/eLife.02763

**Published:** 2014-04-15

**Authors:** Won-Gyu Choi, Simon Gilroy

**Affiliations:** 1 **Won-Gyu Choi** is in the Department of Botany, University of Wisconsin, Madison, United States; 2 **Simon Gilroy** is in the Department of Botany, University of Wisconsin, Madison, United Statessgilroy@wisc.edu

**Keywords:** plant hormone, abscisic acid, FRET-based reporter, biosensors, Arabidopsis

## Abstract

Two independent research labs have developed fluorescent biosensors to report the levels of the stress hormone, abscisic acid, within cells in living plants in real-time.

**Related research article** Waadt R, Hitomi K, Nishimura N, Hitomi C, Adams SR, Getzoff ED, Schroeder JI. 2014. FRET-based reporters for the direct visualization of abscisic acid concentration changes and distribution in Arabidopsis. *eLife* **3**:e01739. doi: 10.7554/eLife.01739; Jones AM, Danielson JÅH, ManojKumar SN, Lanquar V, Grossman G, Frommer WB. 2014. Abscisic acid dynamics in roots detected with genetically encoded FRET sensors. *eLife* **3**:e01741. doi: 10.7554/eLife.01741

**Image** FRET biosensors emit coloured light at different intensities to report the levels of abscisic acid within living plant roots

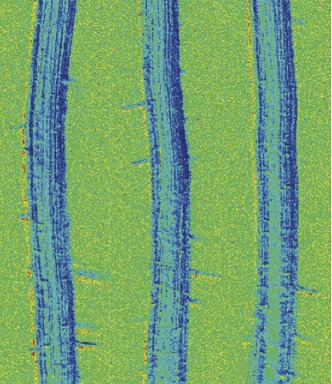

For a plant, the world is full of stress. There are unpredictable rains, freezing winters and baking hot summers. Plants can deal with these insults by detecting when they happen, and then using a suite of hormones to control their own growth and physiology to minimise, or counteract, any damage that might occur. One of the major plant hormones that is involved in the responses to stress, such as drought and heat, is abscisic acid (Hauser et al., 2011). However, monitoring the dynamics of this hormone in living plants, especially at the level of individual cells, had proved very challenging.

Now, in *eLife*, two independent groups of researchers report that they have developed biosensors for abscisic acid, or ABA, that make such measurements a reality. Julian Schroeder of the University of California San Diego and co-workers—including Rainer Waadt as first author—call their bioprobes ‘ABAleons’ (Waadt et al., 2014), while Wolf Frommer at the Carnegie Institution for Science and co-workers—including Alexander Jones as first author—use the name ‘ABACUS’ (Jones et al., 2014). The protein bioprobes developed by the two groups allow researchers to continuously image the levels and movements of abscisic acid in living plants: this is the first time that it has been possible to visualise changes in any plant hormone in this manner.

So why are real-time dynamics important in the apparently slow growing world of plants? ‘Slow growing’ does not mean slow responding. Stress responses that are specifically related to abscisic acid appear throughout the whole plant within 15 minutes of the plant experiencing a heat shock (Suzuki et al., 2012). The signals that triggered these responses must have moved much more quickly, which is why researchers need to be able to make rapid measurements of the signalling dynamics.

As abscisic acid is a small organic molecule, how is it possible to make a biosensor that is able to ‘see’ this hormone? The answer is to combine two advances of modern biology. The first involves technology that is based on a fluorescent protein from a jellyfish: shine a blue light on the jellyfish and it glows green due to fluorescence from the aptly named green fluorescent protein. This protein has been genetically engineered to fluoresce with different colours, and cyan and yellow versions were used for the abscisic acid biosensors. If the cyan fluorescent protein and the yellow fluorescent protein are brought close together, a phenomenon named ‘Förster Resonance Energy Transfer’ (or FRET for short) occurs: in FRET, energy released after exciting only the cyan protein is transferred to the yellow protein, which causes it to emit yellow light (Jones et al., 2013).

To turn this trick of physics into a biosensor involves designing a ‘hinge’ that folds up in response to the factor that you want to detect, and then placing this hinge between the two fluorescent proteins. A hinge that is responsive to, say, calcium ions will bring the cyan and yellow fluorescent protein partners closer together as the hinge folds in response to the binding of calcium ions, and it will move them apart as the hinge unfolds when the calcium ions are released. As such, the relative amounts of cyan and yellow fluorescence given off by the bioprobe reflect the concentration of calcium available within the cell. This technology was indeed first developed for imaging calcium signalling events in mammalian cells and the resulting FRET-bioprobe, named ‘Cameleon’ (Miyawaki et al., 1997), was one of the many reasons why Roger Tsien shared the Nobel Prize in Chemistry in 2008.

In theory, with the correct hinge region, one can build a biosensor for nearly anything that changes in a cell or tissue. So, how could you approach designing the hinge for abscisic acid? The answer to this question awaited our second major scientific advance, this time in the field of plant biology. In 2009, two labs identified a plant abscisic acid receptor (Ma et al., 2009; Park et al., 2009) and found that, once activated, this receptor bound to another protein to trigger the stress responses inside the cell. The Frommer and Schroeder labs realised that by fusing the receptor and its interacting protein side-by-side, they could generate a hinge protein that folds up in response to binding abscisic acid (Figure 1). Using this approach, both groups have generated FRET-biosensors capable of imaging the levels of this hormone. And since the sensors are proteins, both groups have been able to engineer the sensors into living plants. This allows the plants to report the levels of abscisic acid within their own cells, and the movement of this hormone between their cells and tissues, in real-time.

**Figure 1.**
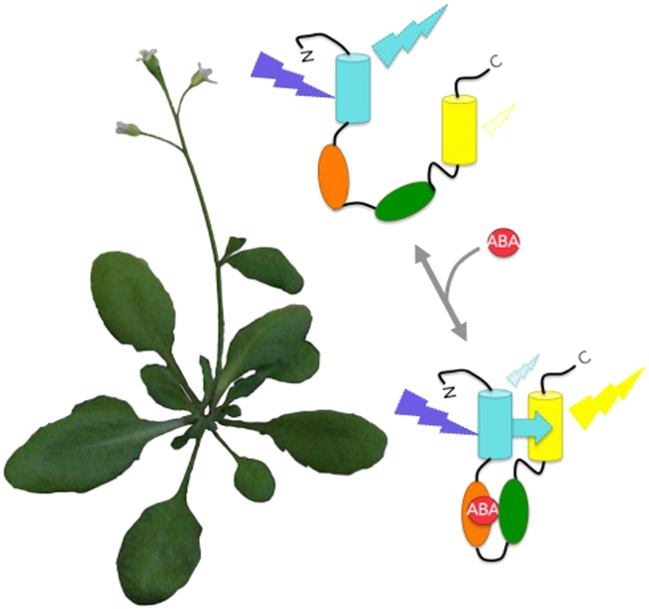
Protein bioprobes allow the imaging of changing abscisic acid (ABA) levels in living plants. The biosensors consist of a single protein containing cyan fluorescent protein, an ABA receptor region, a protein region that naturally binds to the ABA-activated receptor, and yellow fluorescent protein. Cyan fluorescent protein is excited by blue-violet light. For the ABACUS bioprobes, binding to ABA causes the protein to fold up and brings the cyan and yellow fluorescent proteins into close proximity. This increases the efficiency of FRET (cyan arrow), and shifts the colour of fluorescence from more cyan to more yellow (Jones et al., 2014). The ABAleon bioprobes are designed the same way, but work differently in that binding to ABA actually shifts the fluorescence the other way: from more yellow to more cyan (Waadt et al., 2014).

Both groups have already used their FRET biosensors to monitor abscisic acid transport into plant tissues (Jones et al., 2014; Waadt et al., 2014), but this is only the tip of the iceberg that these bioprobes promise to reveal. How do this hormone’s dynamics affect seed maturation and seed dormancy—which are developmental processes closely linked to abscisic acid? Does a wave of this hormone course through the plant as the soil dries? If so, what cell types, or sub-cellular compartments, are involved? These are key questions in stress biology and abscisic acid FRET-bioprobes represent game-changers in our ability to address these questions.
